# Diatom Biosilica Functionalised with Metabolically Deposited Cerium Oxide Nanoparticles

**DOI:** 10.3390/ma17102390

**Published:** 2024-05-16

**Authors:** Izabela Wojtczak, Weronika Brzozowska, Grzegorz Trykowski, Myroslav Sprynskyy

**Affiliations:** 1 Department of Environmental Chemistry and Bioanalytics, Faculty of Chemistry, Nicolaus Copernicus University in Torun, Gagarina 7, 87-100 Torun, Poland; izabelawojtczak1991@gmail.com; 2 Division of Surface Science, Faculty of Chemical Technology and Engineering, Bydgoszcz University of Science and Technology, Kaliskiego 7, 85-796 Bydgoszcz, Poland; weronika.brzozowska@pbs.edu.pl; 3 Department of Materials Chemistry, Adsorption and Catalysis, Faculty of Chemistry, Nicolaus Copernicus University in Torun, Gagarina 7, 87-100 Torun, Poland; tryki@umk.pl

**Keywords:** diatoms, diatom biosilica, cerium(IV) oxide nanoparticles, metabolic insertion, photoluminescence properties

## Abstract

This study introduces a novel approach to synthesising a three-dimensional (3D) micro-nanostructured amorphous biosilica. The biosilica is coated with cerium oxide nanoparticles obtained from laboratory-grown unicellular photosynthetic algae (diatoms) doped metabolically with cerium. This unique method utilises the ability of diatom cells to absorb cerium metabolically and deposit it on their silica exoskeleton as cerium oxide nanoparticles. The resulting composite (Ce-DBioSiO_2_) combines the unique structural and photonic properties of diatom biosilica (DBioSiO_2_) with the functionality of immobilised CeO_2_ nanoparticles. The kinetics of the cerium metabolic insertion by diatom cells and the physicochemical properties of the obtained composites were thoroughly investigated. The resulting Ce-DBioSiO_2_ composite exhibits intense Stokes fluorescence in the violet–blue region under ultraviolet (UV) irradiation and anti-Stokes intense violet and faint green emissions under the 800 nm near-infrared excitation with a xenon lamp at room temperature in an ambient atmosphere.

## 1. Introduction

Cerium oxide nanoparticles are gaining increasing interest in many fields of science and technology, including catalysis, electrochemistry, and medicine [1,2,3] due to their unique properties such as large specific surface area, high surface reactivity, and ability to store and release oxygen [4,5,6]. Cerium(IV) oxide nanoparticles exhibit promising antioxidant properties, enabling their use in medicine. They reduce oxidative stress through the mimetic activity of enzymes such as superoxide dismutase and catalase, regenerating through their ability to switch between Ce^3+^ and Ce^4+^ states [7]. CeO_2_NPs also present neuroprotective potential, allowing their use in the treatment of neurodegenerative diseases such as Alzheimer’s syndrome [8]. Their antioxidant capacity and versatility in targeted drug delivery make them suitable for use in anticancer therapy, where they support the efficacy of drugs such as doxorubicin while minimising their toxic effects on healthy cells [9].

Developing efficient and effective methods for synthesising CeO_2_ nanoparticles, enabling control of their size, shape, and crystallinity, is crucial to optimise their properties and functionality for specific applications. Although many methods for synthesising CeO_2_ nanoparticles have been described in the literature, each has particular advantages and limitations. One commonly used method is the chemical deposition method, which involves the reduction reaction of cerium ions derived from a precursor, which is most often a cerium salt, leading to the formation of CeO_2_NPs. This method allows for the relatively simple and inexpensive production of cerium(IV) oxide nanoparticles with a large specific surface area. However, it can lead to nanoparticles of variable homogeneity and size, as confirmed by the work of Chen et al. [10], which describes using a chemical deposition method to synthesise CeO_2_ nanoparticles with high catalytic activity in nitrogen oxide reduction processes. Based on the hydrolysis and polymerisation of cerium precursors, the sol–gel method allows precise control over the morphology and size of nanoparticles, which is crucial for their use as substrates for the production of catalysts or biosensors. The work of Li et al. [11] demonstrates the application of this method to the synthesis of CeO_2_ nanoparticles acting as catalysts in the degradation of 2,4-dichlorophenoxyacetic acid, with the ability to manipulate the morphology and size of these particles. On the other hand, although often more complicated and costly, physical methods such as thermolysis or solvothermal methods make it possible to obtain CeO_2_ nanoparticles with a highly pure crystal structure and a high homogeneity. By adjusting the conditions of the synthesis reaction, such as solvent composition, precursor concentration, temperature, and time, CeO_2_ nanoparticles of different shapes and adjustable size can be obtained, as shown in the work of Wu et al. [12], which described the use of a thermolysis process to obtain uniform CeO_2_ “nanoflowers”. Also, the hydrothermal method, widely discussed in the literature, allows for the efficient synthesis of cerium(IV) oxide nanoparticles, providing control over their morphology and crystalline properties, as demonstrated in the work of Zhang et al. [13], where an environmentally friendly method for synthesising CeO_2_ hollow spheres with a crystalline mesoscopic coating is described. The choice of an appropriate method for synthesising CeO_2_ nanoparticles depends on several factors, including the specific properties of the final product, the scale of production, and cost.

This study presents the results of the diatom biosilica functionalisation study using metabolically deposited cerium oxide nanoparticles. We propose an innovative, eco-friendly approach based on biosynthesis involving unicellular microorganisms [14] such as diatoms, whose cells can incorporate and deposit cerium from the growing medium. The resulting 3D micro-nanostructured amorphous silica coated by cerium oxide nanoparticles combines the unique properties of diatom biosilica and cerium oxide nanoparticles.

## 2. Materials and Methods

### 2.1. Biomass Cultivation of Diatom Species Pseudostaurosira trainorii

A strain of *Pseudostaurosira trainorii* diatoms was obtained from the Baltic Algae Culture Collection of the Institute of Oceanography of the University of Gdansk. These microorganisms were carried out in Erlenmeyer flasks with a capacity of 5000 mL at a constant temperature of about 25 °C. Guillard F/2 medium was used to ensure optimal culture conditions, and sodium metasilicate (Na_2_SiO_3_ × 5H_2_O) was added to provide a soluble silicon source. Additionally, the medium was enriched with cerium ions using ammonium cerium(IV) nitrate. The range of used silica concentration in the second experiment series was 7, 17.5, 35, 52.5, 61.25, 70, and 78.75 mg Si/L. The concentration of cerium in the third experiment series was 7, 14, 21, 28, 35, 43.75, 52.5, 61.25, 70, and 78.75 mg Ce/L, depending on the experiment. To maintain optimal conditions, a constant pH level was monitored, continuous aeration was provided, and controlled lighting was used to simulate the natural cycle of day and night (12 h of light and 12 h of darkness), using two 1500 lux light sources. The entire culture process, lasting 12 days, aimed to produce diatom biomass of the selected diatom species.

### 2.2. Procedure for Cleaning Diatom Frustules

Once the diatom culture was completed, the filtered biomass was purified to isolate the diatom biosilica. This step occurred in 600 mL flat-bottomed beakers with a 30% hydrogen peroxide solution. The process required maintaining a constant temperature of 80 °C and stirring with a magnetic stirrer for 4 h. After this, a few drops of 37% hydrochloric acid (HCl) were added to the beaker to remove calcium carbonates and excess hydrogen peroxide. Then, the purified biosilica was rinsed with distilled water and left for 24 h to allow sedimentation of the residue. The next step was decantation—carefully pouring off the liquid without disturbing the sediment at the bottom of the vessel. Then, the material was centrifuged in an Eppendorf Centrifuge 5810 R at 10,000 rpm for 10 min and decanted. The centrifugation process was repeated five times with distilled water added. Finally, the obtained diatom biosilica (DBioSiO_2_) was dried at 120 °C and stored in sterile Eppendorf tubes after drying.

### 2.3. Methods of the Material Characterisation

Detailed morphological analysis of the materials obtained was possible through advanced electron microscopy techniques. These studies used a Quanta 3D FEG SEM/FIB scanning electron microscope with an secondary electrons (SE) signal detector. This apparatus allows for high-resolution images of up to 1.2 nm (FEI, Boynton Beach, FL, USA). Before analysis, material samples were subjected to a nanometre-thin gold coating process, which was necessary to obtain clear and precise images. This procedure was carried out under variable vacuum conditions, which is crucial to preserve the integrity of the sample and the quality of the data obtained. To determine the elemental composition of the materials analysed, a scanning electron microscope (SEM, LEO 1430 VP, Electron Microscopy Ltd., Cambridge, UK) coupled to an energy dispersive X-ray detector (XFlash 4010, Bruker AXS, Bremen, Germany) was used.

Microscopic measurements were carried out using a Tecnai Osiris transmission electron microscope (FEI, Hillsboro, OR, USA). A dark field (DF) detector was used for visualisation in transmission electron microscopy-TEM mode. In Scanning transmission electron microscopy STEM mode, a high-angle annular dark field (HAADF) detector was used for imaging purposes, and a Super-X 130 eV energy dispersive spectrometer (EDS) was used for elemental analysis. The sample preparation procedure involved the following steps: first, a few milligrams of the substance were dissolved in ethanol (99.8% anhydrous) and ultrasonicated for 5 s; second, 5 μL of this solution was applied to a copper grid covered with a carbon layer containing holes (Lacey Cu 400 mesh type); third, after evaporation of the solvent at room temperature, the dry powder remaining on the grid was the object of study.

Cerium uptake by diatom cells was controlled using a Shimadzu ICP-MS 2030 inductively coupled plasma mass spectrometer (Kyoto, Japan). The 5 mL samples containing the culture medium were collected each day for 12 days of cultivation. Collected samples were then diluted 100-fold in ultrapure 1% HNO_3_.

The crystal structure and phase composition of the sample were analysed using X-ray powder diffraction (XRD). A Philips X’Pert Pro diffractometer (Malvern Panalytical Ltd., Malvern, UK) was used for experimental purposes, using Cu-Kα radiation with a wavelength of λ = 0.1541 nm. XRD measurements were carried out using an anode voltage of 40 kV and a current of 30 mA, providing optimal conditions for precise analysis results. Data were collected for angle values 2θ from 10° to 90° with the measurement step at 0.01°.

The surface potential of the materials (Zeta-ζ potential value) was measured using a Zetasizer Nano Series instrument (Malvern Instruments, Malvern, UK). Before measurement, the analysed samples at a concentration (0.05 mg/mL) were suspended in water at pHs of 2.03, 3.01, 5.04, 8.02, 10.02, and 12.00. In addition, the samples were sonified in a Polsonic ultrasonic bath for 360 min at 25 °C. A DTS1070 measuring cell was used to measure the zeta potential. The measurements included three repetitions at the above pH values for each sample analysed.

The thermal stability of the synthesised materials was analysed using a Netzsch Jupiter STA 449 F5 thermoanalyzer (NETZSCH Analyzing & Testing, Burlington, MA, USA) at temperatures from 29 °C to 1050 °C. The samples were heated at 10 °C min^−1^ in a nitrogen atmosphere.

A detailed analysis of structural bonds and functional groups was carried out in the studied materials using advanced spectroscopic techniques. For this purpose, a Fourier transform infrared (FTIR) spectrophotometer was used (Fourier Transform Infrared Spectroscopy using Attenuated Total Reflectance-FTIR ATR model, Vertex 70, from Bruker Optik, Billerica, MA, USA). This instrument has a DLaTGS (deuterated L-alanine doped triglycene sulphate) detector, which provides high sensitivity and precision measurements. The procedure for recording FTIR spectra was carried out with great care, which included averaging scans collected over a wide range of wavenumbers, from 400 cm^−1^ to 3500 cm^−1^. The chosen measurement resolution was 4 cm^−1^, which made it possible to obtain the detailed and precise spectra necessary to identify the structures under study accurately.

As part of the analysis of the optical properties of solid samples, a Jasco V-750 spectrophotometer (Jasco, Hachioji-shi, Japan) was used to measure the absorption spectrum in the UV-Vis range. Measurements were carried out in the wavelength range from 250 nm to 900 nm, using normal incident light, which allowed detailed absorption characterisation of the materials under study. In addition, a Hitachi F-2500 fluorescence spectrophotometer (Hitachi, Chiyoda, Japan), equipped with a xenon lamp, was used to study photoluminescence (PL) properties. PL spectra were recorded at room temperature, i.e., about 20 °C, under ambient atmosphere conditions. The analysis was conducted for solid samples (DBioSiO_2_ and 10%Ce-DBioSiO_2_) placed in a specially adapted cuvette. Slits of 2.5 nm width were used for the measurements, with a lamp voltage of 700 V and a scanning speed of 300 nm/min. Photoluminescence measurements were performed at different excitation wavelengths: 270 nm, 420 nm and 800 nm, making it possible to comprehensively analyse the test analyte’s emission properties.

The study involved the determination of the fluorescence quantum yield using a relative method for a 10% Ce-DBioSiO_2_ sample. A vital element of this method is the comparison of the unknown quantum yield of the sample under study with the quantum yield of a well-defined standard. This process requires ensuring the same absorption of light of a specific wavelength by the analysed sample and the reference solution, which is the foundation of the relative method. For calculation purposes, the quantum yield of the sample relative to the standard is determined by comparing the integrated fluorescence spectra of the two solutions, measured under identical conditions. The calculation also takes into account the refractive indices of the solvents if the sample and standard are in different media, as expressed in the equation below:

(1) $$n_{{fl}_{sample}}=n_{{fl}_{reference}}\times \frac{{IF}_{sample}}{{IF}_{reference}}\times \frac{n_{sample}^{2}}{n_{reference}^{2}}\;$$

To determine the fluorescence quantum yield, a solution of the sample in 96% ethanol (Merck, Darmstadt, Germany) was prepared, and a standard solution of quinine bisulphate (Satna Cruz Biotechnology, Heidelberg, Germany) in 0.5 M sulfuric acid(VI) (Merck, Darmstadt, Germany) was used. Fluorescence measurements for the sample and standard were made using a Hitachi F-2500 fluorescence spectrophotometer at an excitation wavelength of 370 nm. Both the sample and the standard had identical absorption at 0.14. These measures made it possible to determine the area under the integrated fluorescence spectra, which formed the basis for calculating the fluorescence quantum yield value according to Equation (1).

## 3. Results and Discussion

### 3.1. Kinetics of Cerium Sorption by Diatom Cells from the Culture Medium and Dependence on the Harvested Diatom Biomass on the Concentration Ratio Si/Ce in Culture Medium

**Figure 1:**
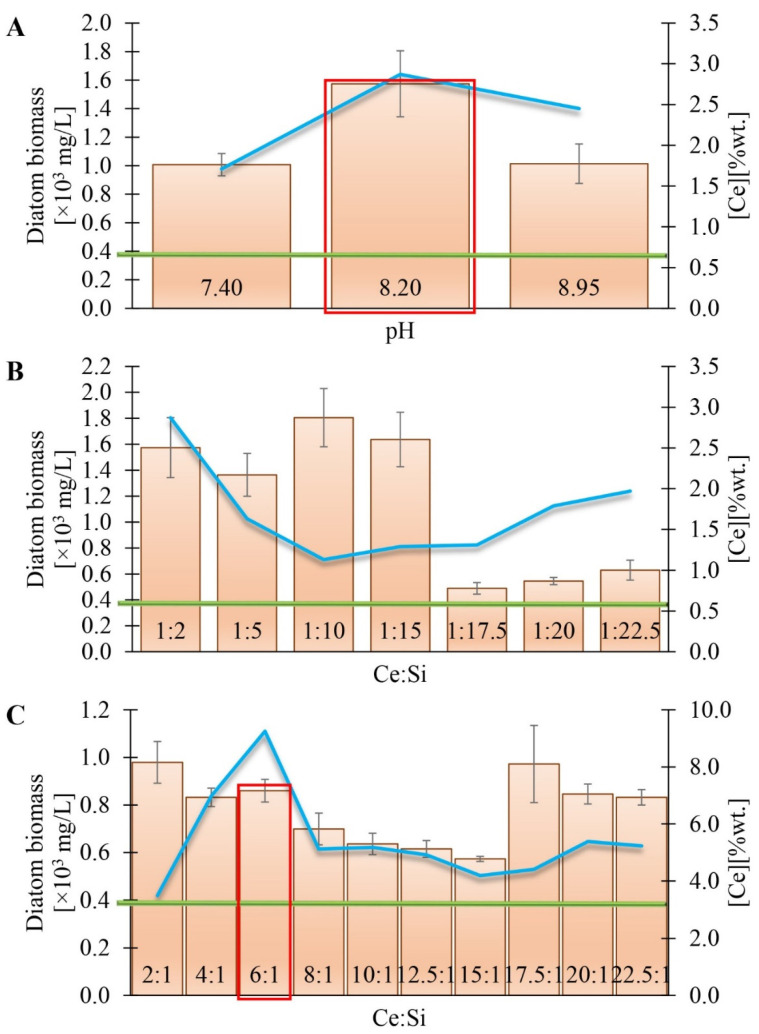
The amount of biomass obtained on the last day of the experiment depends on (**A**) the pH value and (**B**,**C**) the Ce:Si ratio of the content (%wt.) of cerium in diatom biomass (blue line). Key experiments for each series were marked with a red rectangle. Biomass accumulation of unmixed diatom cells (green line).

Figure 1 shows data on the balance of cerium incorporation into diatom cells and the amount of diatom biomass harvested on the last day of culture. These studies were carried out in three experimental series (Figure 1A–C). McFarland measurements were used to standardise cerium-doped biomass using the appropriate conversion factor determined in earlier works [15,16]. The process’ yield of 0.39 mg/L of undoped diatom cells (green line) served as a reference to determine the effect of individual factors on the amount of biomass harvested.

The experiment shown in Part A (Figure 1) analysed the effect of pH changes on the amount of diatom biomass harvested. Most diatom species prefer a culture medium whose pH oscillates between seven and nine [17,18]. This suggests that optimisation of this value is crucial in the process of efficient diatom cell proliferation. This is likely because an appropriate pH value significantly impacts maintaining the efficiency of biological processes at the cellular level [18]. Accordingly, the pH values adopted for Experiment A are 7.40, 8.20, and 8.95. The initial concentration of soluble silicon species was 7.0 mg Si/L, while the initial concentration of soluble cerium was 3.5 mg Ce/L.

Analysing the results shown in Figure 1A, we noticed that, regardless of the pH value of the culture medium, the amount of biomass on the last day of culture exceeded the reference value (which was true for all series). In addition, an increase in the amount of biomass obtained was observed as the pH value of the culture medium increased until a limiting pH value of 8.2 was reached, after which there was a decrease in the number of cells in the biomass. It follows that the optimal pH value, leading to the maximum efficiency of the culture process, is 8.2. The results confirmed the data from previous literature [19], which considered pH values from 8.2 to 8.9 optimal for microalgae culture. In addition, we noted that, as the pH increased to a value of 8.2, the amount of incorporated cerium in the diatom cells increased. For this reason, the value of pH = 8.2 was chosen for further experiments as the most optimal value, combining the highest efficiency of the biomass culture process with the most significant amount of incorporated cerium into the diatom cells.

Figure 1B,C illustrate the results of experiments that analysed the effects of different Ce:Si ratios on the performance of the diatom biomass culture process. The series shown in Figure 1B consisted of seven tests in which the Ce:Si concentration ratios were, respectively, 1:2, 1:5, 1:10, 1:15, 1:17.5, 1:20, and 1:22.5. The initial concentration of soluble cerium was 3.5 mg Ce/L and was a constant value in all tests. The concentration of soluble silicon in the culture medium varied depending on the tests, ranging from 7 mg Si/L to 78.75 mg Si/L. The pH value of the culture medium was 8.2 for all tests. The choice of pH values was based on the results of experiment 1A. Analysing the test results presented in Figure 1B, we noted that an increase in silicon concentration in the medium increases the amount of collected biomass. This increase occurs until a limiting concentration is reached at a Ce:Si ratio of 1:20, above which a decrease in the efficiency of the culture process is observed. This suggests a specific permissible concentration of silicon, which, if exceeded, reduces the amount of incorporated silicon in the diatom frustule and, consequently, decreases the number of cells in the diatom biomass [20]. In addition, the results obtained in Experiment B indicate that a direct correlation between an increase in silicon concentration and increased cerium uptake by diatoms is impossible to observe. This shows the limited ability of cells to incorporate cerium at high silicon concentrations in the culture medium.

Figure 1C presents the results for ten Ce: Si mass ratios, where cerium was the dominant concentration relative to silicon. The values of these ratios were 2:1, 4:1, 6:1, 8:1, 10:1, 12.5:1, 15:1, 17.5:1, 20:1, and 22.5:1, respectively. The initial concentration of soluble silicon in the culture medium was 3.5 mg Si/L and was constant for all tests. The initial concentration of soluble cerium varied between assays, ranging from 7 mg Ce/L to 78.75 mg Ce/L.

We observed that the amount of biomass obtained decreases as the concentration of cerium increases in the culture medium, which is consistent with the results obtained in Experiment B. This decrease in biomass obtained is noticeable until the initial concentration of cerium in the culture medium is 52.5 mg Ce/L. Exceeding this value results in a sharp increase in biomass obtained (Ce:Si, 17.5:1), followed by another decrease. Analysis of the results on the content of incorporated cerium depending on its concentration in the culture medium shows that as the amount of cerium in the medium increases, the amount of cerium absorbed by the cells increases, which occurs until the concentration of cerium in the medium reaches 21 mg Ce/L. At this point, the cerium content of the diatom cells reaches a maximum (9.25 wt.%). An increase in the concentration of cerium in the medium to 52.5 mg Ce/L results in a decrease in the amount of cerium incorporated into the diatoms, evident until the initial concentration of Ce increases to 70 mg Ce/L. At this point, a slight increase in cerium content in diatom cells is again observed.

The above experiments on optimising the pH and cerium/silicon concentration ratio resulted in a representative material that compromises the amount of biomass obtained and the cerium content of the diatom cells. This selected material is biomass doped with cerium ions with an initial concentration of 21 mg Ce/L (with an initial silicon concentration of 3.5 mg Si/L), subjected to further analysis, the results of which are described in this article.

**Figure 2:**
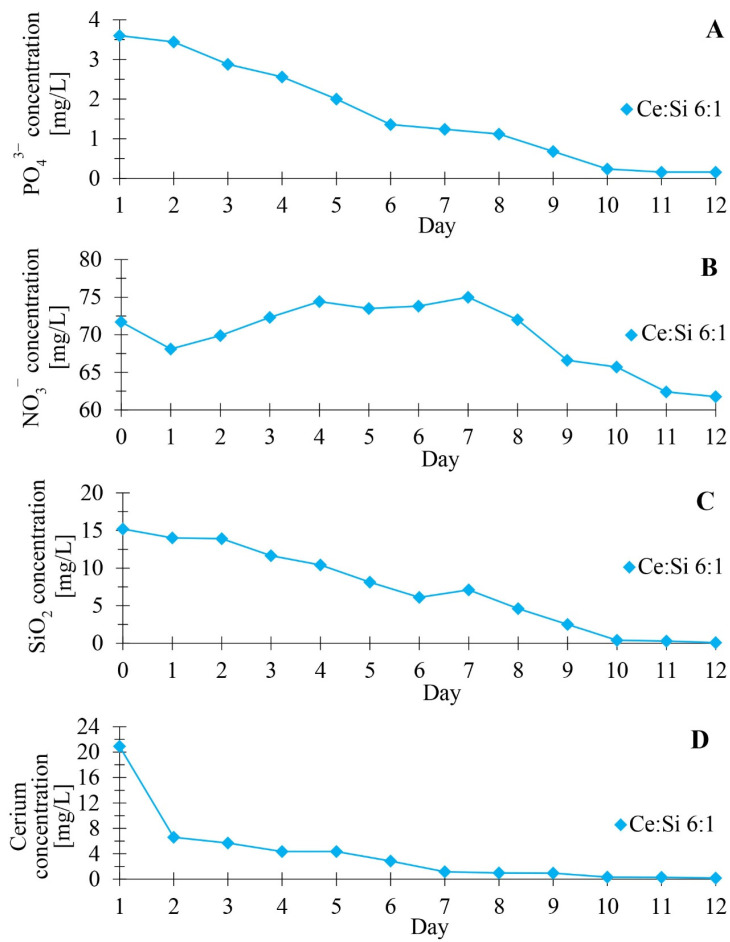
Kinetics of cerium and nutrients uptake by diatom cells from the culture medium: (**A**) phosphorus, (**B**) nitrogen, (**C**) silicon, and (**D**) cerium.

Figure 2 presents the changes in the concentration of phosphate, nitrate, silica, and cerium during a 12-day culture of diatoms doped with cerium ions with an initial concentration of 21 mg Ce/L. The content of the components mentioned above in the culture medium was examined using photometric assays and the ICP-MS technique. Analysis of the obtained data on the rate of nutrient uptake from the culture medium showed that an intensive uptake of phosphorus, nitrogen, silicon, and cerium occurs during the first few days of culture. These observations correlate with the data in the literature, emphasising that microalgae’s rapid growth phase is often observed early in their development [21,22]. During this period, nutrient availability is not a limitation to the intensification of microalgae biological processes. Over time, as the rate of cell division of microalgae decreases, their biomass accumulates, which continues until nutrients in the medium begin to limit the growth of diatom cells, leading to the stationary phase.

Figure 2A illustrates the gradual decrease in phosphate concentration, which reaches a value below the detection limit at the end of the culture period (a drop in concentration from about 4 mg/L to near zero on day 12). Figure 2B presents nitrate concentration changes, which remain stable at around 70 mg/L for most of the period analysed. The stability of the nitrate concentration may be due to the specificity of the cerium complex used. Figure 2C shows the change in the concentration of silica, a key ingredient necessary for synthesising diatoms’ cell walls. The systematic decrease in SiO_2_ concentration may indicate active growth and intensive construction of new cells. Figure 2D presents the uptake of cerium by diatoms, where a marked decrease in the concentration of this element was observed after the sixth day of the experiment. The coincidence of the absorption dynamics of cerium with silicon suggests analogous mechanisms of uptake of these elements by diatom cells.

### 3.2. SEM and SEM-EDX Studies of Synthesised Composites

**Figure 3:**
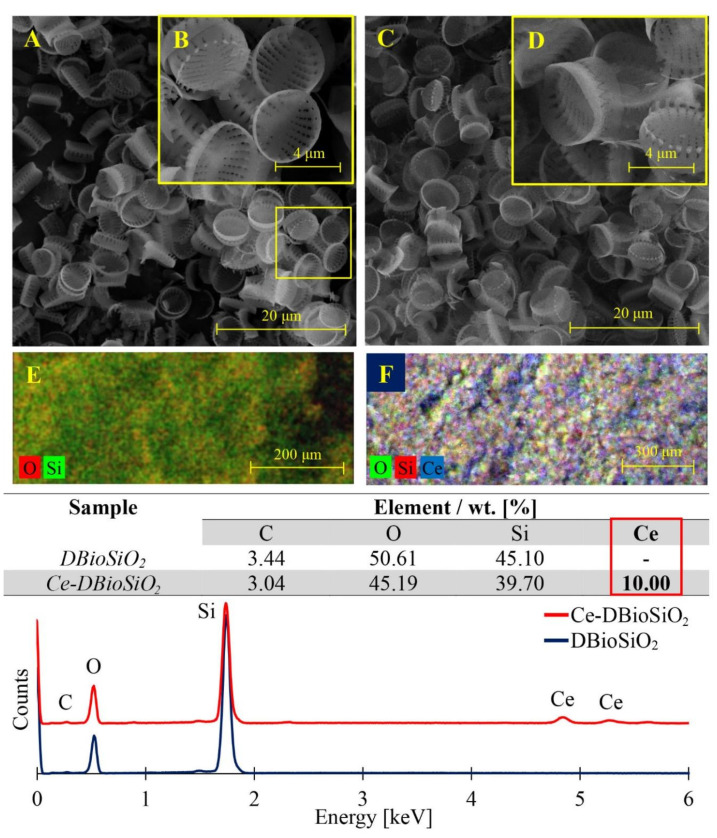
Scanning electron microscopy images of pure diatom biosilica (**A**,**B**) and biosilica doped with cerium (**C**,**D**), together with the results of analysis of elemental composition and their distribution (**E**,**F**) in the analysed materials.

The results in Figure 3 indicate an overview of SEM microphotographs and EDX analysis results of pure biosilica derived from the diatom species *Pseudostaurosira trainorii* and biosilica doped with cerium ions. Comparing the morphology of cerium ion-modified frustules (Figure 3C,D) with undoped frustules (Figure 3A,B), it can be seen that despite the modification processes, the intricately decorated, three-dimensional structure of diatom frustules was preserved, along with its symmetry and regularity of structural elements [15,23]. This indicates the high structural stability of the diatom frustules. SEM-EDX analysis confirmed that pure diatom frustules are composed of oxygen and silicon, while frustules doped with cerium ions are composed of oxygen, silicon, and cerium.

The presence of Si, O, and Ce in the modified diatom shells indicates that the cerium remains bonded to the surface of the diatom frustule despite etching with H_2_O_2_ and HCl solutions. This bonding is likely due to combining biosilica silanol groups with cerium oxides. The cerium content of the Ce-DBioSiO_2_ sample was 10% by weight, which determines the terminology of the doped sample used later in the paper (10%Ce-DBioSiO_2_). The small amount of carbon in both materials may be related to residual organic materials in the biosilica structure and the high background noise level for carbon in SEM-EDX. Figure 3E,F show distribution maps of the elements of the analysed materials. Figure 3E shows the distribution of silicon and oxygen in pure diatom biosilica, while Figure 3F illustrates the distribution of silicon, oxygen, and cerium in a biosilica sample doped with cerium ions. It should be noted that in both cases presented, the distribution of the elements above is uniform and reproducible.

### 3.3. Transmission Electron Microscopy Study

**Figure 4:**
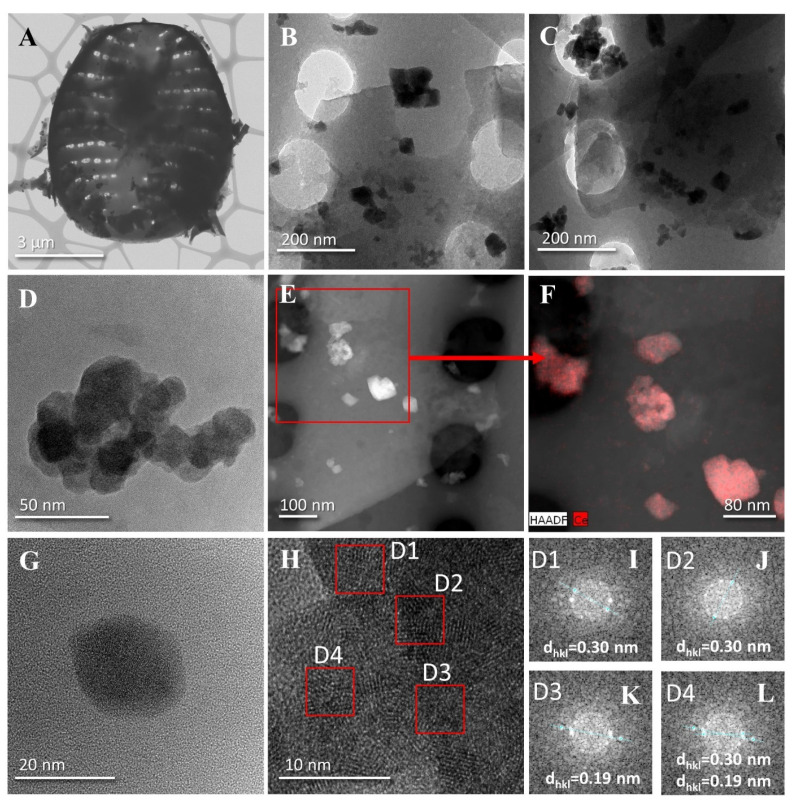
TEM images of the composite 10%Ce-DBioSiO_2_: (**A**)—a single frustule covered with cerium nanoparticle clusters, (**B**,**C**)—magnifications of selected fragments of the diatom frustules, (**D**)—magnification of the agglomerate/cluster of the created nanoparticles, (**E**)—STEM image of NPs distribution on the frustule, (**F**)—TEM-EDX mapping of cerium distribution, (**G**)—HR-TEM view of a single cerium nanoparticle, (**H**)—HR-TEM image of cerium nanoparticles, and (**I**–**L**)— selective area electron diffraction (SAED) of individual nanoparticles.

Figure 4 presents a detailed overview of the morphology and structure of diatom biosilica doped with cerium (10%Ce-DBioSiO_2_). Figure 4A illustrates the complex structure of diatom frustules. The architecture of the entire diatom shell is visible, as well as the details of the ordered pore network structure [23,24]. We noted that adding cerium to DBioSiO_2_ does not exhibit a toxic effect on the shape and structure of diatom frustules. Similar to the work by Bour et al., no effects of deformation or distortion of the diatom shell surface were observed, which could lead to irregular structure [25]. Figure 4B,C,E,F depict the distribution of the created cerium nanoparticles, exhibiting an irregular, quasi-cubic shape. The cerium nanoparticles are uniformly distributed throughout the biosilica matrix. The presence of nanoparticles was confirmed throughout the frustule, on its edges, in the central part, and the pores. Figure 4D presents a typical nanoparticle aggregate on the biosilica surface, where individual crystalline forms can be observed. The size of the aggregates appearing on the surface of the frustules is around 100 nm. Similar results were described by Qian et al., who obtained aggregates measuring about 100 nm in size, consisting of many mutually connected cerium nanoparticles [26]. The tendency for self-organisation into larger structures is a typical phenomenon among nanoparticles, often attributed to strong van der Waals interactions [27]. Nanoparticles with a small size and a large specific surface area have a very large uncompensated surface energy. They tend to reduce this energy through aggregation, a thermodynamically favourable process. Furthermore, the surface of the formed nanoparticles serves as a substrate for the nucleation and growth of subsequent nanoparticles. Figure 4G presents a single, well-defined, quasi-cubic ceria nanoparticle with a size of 20 nm. In our previous work, using the chemical reduction of cerium ion precursor, we obtained CeO_2_ nanoparticles with sizes ranging from 3–5 nm [28].

Figure 4H presents an high-resolution transmission electron microscopy HR-TEM image of octahedral cerium nanocrystals [29], which shows the lattice striations of CeO_2_. The crystal structure of the CeO_2_ nanoparticles, along with the determined distances between the planes, is shown in Figure 4I–L. A lattice stripe spacing of 0.3 nm corresponds to the d-plane spacing {111} [30], while a lattice stripe spacing of 0.19 nm characterises the {220} crystal plane [31]. These values are characteristic of the face-centred cubic fcc structure of CeO_2_ [32].

### 3.4. Powder X-ray Diffraction Analysis

**Figure 5:**
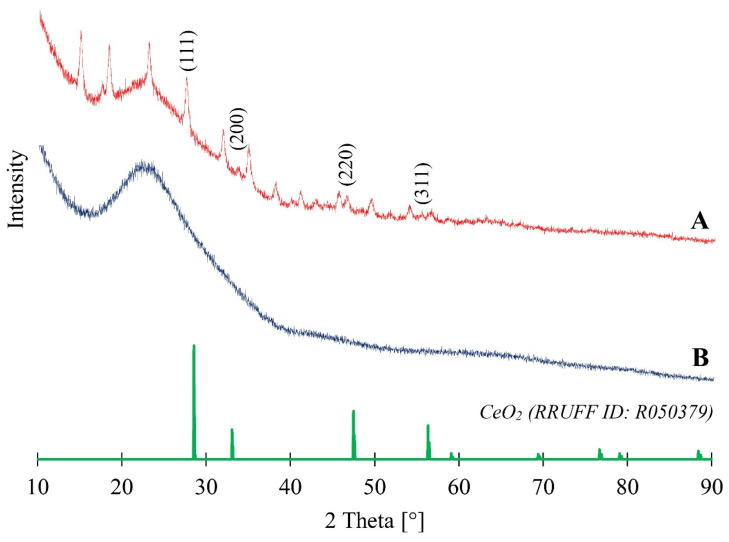
XRD patterns of 10%Ce-DBioSiO_2_ composite (**A**) and pure diatom biosilica DBioSiO_2_ (**B**).

Figure 5 presents the results of the X-ray structural analysis of two samples: sample A (biosilica doped with CeO_2_NPs—10%Ce-DBioSiO_2_) and sample B (undoped biosilica from the diatom species *Pseudostaurosira trainorii*—DBioSiO_2_). In both samples, a broad peak was observed from 15° to 30° 2θ angle, corresponding to the amorphous form of silica, represented by opal-A (RRUFF ID: R060653). These results are consistent with the conclusions described by Sprynskyy et al. regarding biosilica obtained from the diatom species *Pseudostaurosira trainorii* [15]. The XRD results for sample A indicate that it is possible to determine peaks for 2θ angle values of 27.36°, 33.57°, 46.40°, and 56.52°. The unidentified diffraction peaks do not match typical peaks for silicates, carbonates, or hydroxides and do not find equivalents in existing databases.

Comparing the XRD spectrum of CeO_2_ nanoparticle-doped biosilica with the reference spectrum reveals slight shifts in the 2θ values of characteristic peaks of the crystalline form relative to the reference substance. These changes are likely due to the resulting material complexity. Understanding how nanoparticle size and material structure complexity affect XRD diffractograms in the context of nano-scale material characterisation via powder X-ray diffraction is essential. Crystal size reduction leads to the broadening of the diffraction peaks, as described by the Scherrer equation, which affects the precision of determining the 2θ values, which is critical in nanoparticle analysis. The preferential orientation of particles, resulting from their irregular shapes, is another factor that can impact the intensity and location of peaks in the XRD diffractogram. The structural complexity of the material under study is an additional complicating factor in interpreting XRD results. Comparing XRD results with simulated or standard diffraction patterns can help identify crystalline phases. Still, one must know potential differences from factors like particle size or orientation [32].

The X-ray diffractogram obtained for the Ce-DBioSiO_2_ composite shows a slight shift of diffraction peaks in the 2θ values compared to those presented for the standard. This may be due to the shrinking of the CeO_2_ network, which is associated with changes in the CeO bond length and generated distortions. This assumption aligns with literature reports describing, for instance, doping of CeO_2_NPs with other elements like cobalt, which led to deformations in the crystal lattice of the nanoparticles, consequently affecting the unit cell parameters and resulting in shifted peaks towards higher 2θ values [33,34]. The likely cause of the crystal lattice deformation in the obtained CeO_2_ nanoparticles could be their synthesis on a complex matrix (hydrated amorphous biosilica), the cleaning process (using H_2_O_2_ and HCl), and the application of temperature (during material drying). Studies of the H_2_O_2_ adsorption and decomposition on various CeO_2_ (111) surface models showed that hydrogen peroxide adsorbed on the ceria surface may heterolytically dissociate to form two hydroxyl groups at two vicinal Ce sites [35]. The hydrogen peroxide adsorption also affects the concentration of Ce^3+^ on the CeO_2_ surface [36]. The generation of additional hydrogen bonds with the formation of hydroxyl groups and oxygen vacancies on the CeO_2_ surface under the influence of H_2_O_2_ (used to purify biosilica) is reflected to some extent in the structural parameters of the formed cerium oxide nanoparticles. The significant increase in hydration of cerium-doped biosilica compared to pure biosilica, detected by the thermogravimetric analysis data (see below), may be due to the same reasons.

### 3.5. Thermogravimetric Analysis

**Figure 6:**
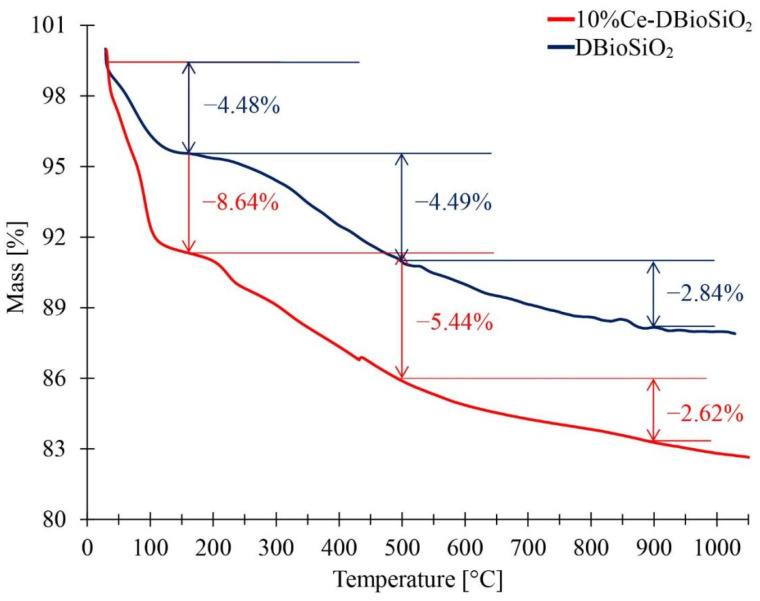
The thermogravimetric curves of DBioSiO_2_ (dark blue line) and 10%Ce-DBioSiO_2_ (red line) samples.

Figure 6 presents the results of thermogravimetric (TG) analysis for a sample of diatom biosilica (DBioSiO_2_) and a sample of cerium-doped material (10%Ce-DBioSiO_2_). TG analysis was conducted in a nitrogen atmosphere to assess these materials’ thermal stability and degradation mechanisms in the temperature range from 29 °C to 1050 °C. For both analysed samples, three stages of mass loss can be observed, occurring at the following temperature ranges: stage I—from 29 °C to 160 °C, stage II—from 160 °C to 500 °C, and stage III—from 500 °C to 900 °C. The first stage of mass loss in the samples involves their dehydration, i.e., the removal of physically bound water molecules from the surface of the samples.

The remaining two stages of mass loss result from the dehydroxylation process, where -OH groups are released. This leads to the formation of hydrogen bonds between oxygen atoms, resulting in the formation of water molecules, which then evaporate. In the case of siliceous materials, this process typically involves breaking Si-OH bonds, leading to condensation and the formation of new Si-O-Si bonds [37,38]. The total mass loss of the DBioSiO_2_ sample is 11.81%. In comparison, the total mass loss of the material with cerium addition is 16.70% (up to 900 °C), indicating the high thermal stability of both materials at elevated temperatures. During dehydration, 10%Ce-DBioSiO_2_ shows a more significant mass loss (8.64%) than the undoped biosilica (4.48%). This likely results from the complex structure of the doped material, where interactions between CeO_2_ nanoparticles and the biosilica matrix may provide additional sites for water adsorption, thereby causing more significant mass loss during the initial heating stage [39,40]. Conducting the calcination process in the 400–800 °C temperature range may lead to well-crystallized CeO_2_ nanoparticles characterised by hydrophobicity [41,42]. CeO_2_NPs can alter the adsorption and desorption properties of the materials containing them, directly influencing mass loss during sample heating. In materials with crystallised CeO_2_NPs, mass loss associated with dehydration may occur more intense than in materials without cerium dopants [43,44,45,46], as observed in Figure 6. At higher temperatures, the mass loss is similar for both samples, amounting to 2.84% for DBioSiO_2_ and 2.62% for 10%Ce-DBioSiO_2_.

### 3.6. Zeta Potential Measurements

**Figure 7:**
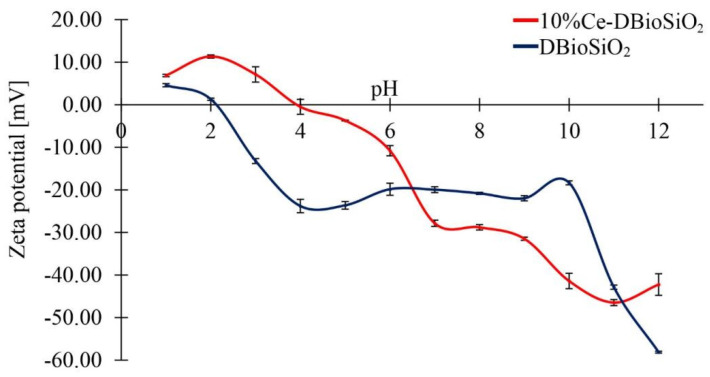
Dependence of zeta potential values on pH for DBioSiO_2_ (dark blue line) and 10%Ce-DBioSiO_2_ (red line).

The electrokinetic stability of the material was evaluated by measuring the zeta potential (ζ-potential) in the pH range from one to twelve. Figure 7 shows the dependence of the zeta potential value on pH for pure biosilica and biosilica doped with cerium. In the pH range studied, the zeta potential value decreases with increasing pH for both samples (from +6.89 mV at pH = 1 to −58.11 mV at pH = 12). This decrease may be due to the progressive deprotonation of silanol groups (Si-OH ↔ SiO^−^ + H^+^), which leads to an increase in the negative surface charge of the particles [47].

The addition of cerium to the biosilica leads to a decrease in its zeta potential, particularly evident for pH > 6. In addition, it is worth noting that the isoelectric point (IEP) for pure biosilica is located at pH = 2. This is a direct effect of the polar measurement environment [48], which causes the surface silanol groups to have acidic properties. The addition of cerium ions shifts the IEP value to pH = 4, which results from a change in the surface charge of the biosilica after the adsorption of cerium(IV) oxide nanoparticles [49]. In addition, the IEP of doped material localised at pH ~four is characteristic of CeO_2_ nanoparticles obtained by chemical synthesis methods [49]. Above pH values of 10, a significant decrease in the zeta potential value was observed for all tested materials, probably due to the deprotonation of all hydroxyl groups on the surface of diatom shells [50]. The increase in the negative zeta potential value of biosilica doped with CeO_2_ nanoparticles compared to pure biosilica may be due to the larger specific surface area of biosilica due to the presence of nanoparticles.

### 3.7. FTIR Spectra

**Figure 8:**
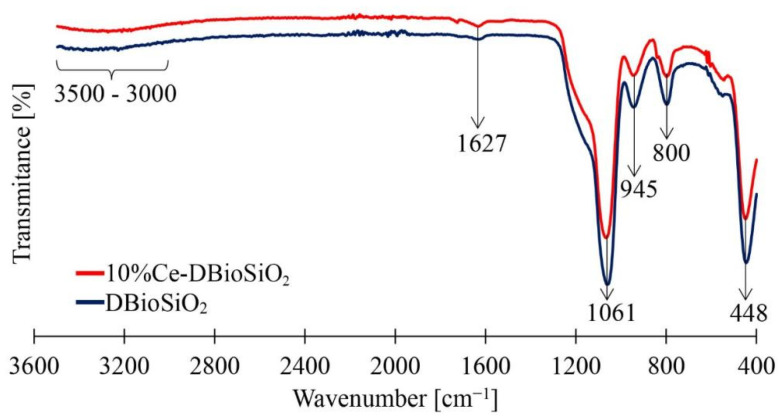
FTIR spectra of undoped biosilica (dark blue line) and biosilica doped with cerium ions (red line).

Figure 8 presents FTIR spectra of biosilica doped with cerium ions (10%Ce-DBioSiO_2_) and undoped biosilica (DBioSiO_2_). Both spectra contain characteristic absorption bands at 448, 800, and 1061 cm^−1^ associated with valence and deformation vibrations of the Si-O-Si siloxane bonds present in the amorphous silica [51,52].

In the case of doped material, the absorption peak maximum at 448 cm^−1^ may additionally result from Ce-O bonds in the CeO_2_ phase [53]. Absorption bands at 1627 and 3000–3500 cm^−1^ can be identified in both spectra, resulting from valence and O-H deformation vibrations occurring in molecular water and hydrated forms of biosilica. An absorption band at 945 cm^−1^ was observed in both spectra, indicating the presence of a Si-OH silanol bond, typical of marine diatoms [54].

### 3.8. Photoluminescence Properties

**Figure 9:**
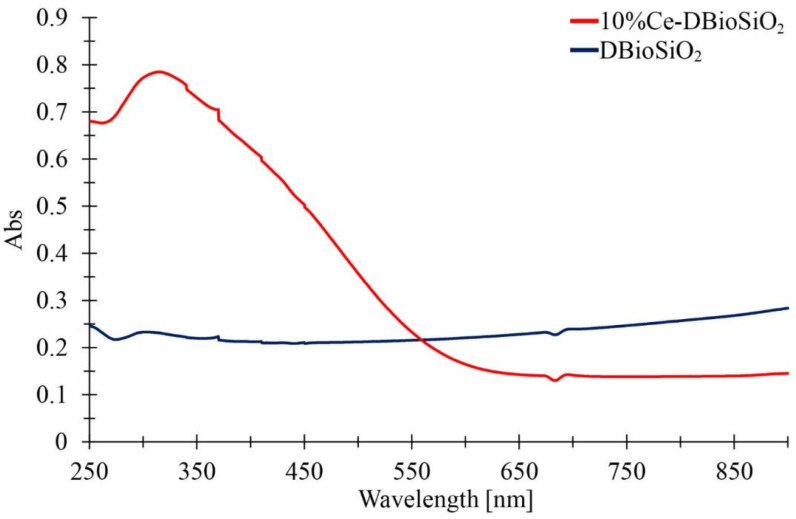
UV-Vis absorption spectra of DBioSiO_2_ and 10%Ce-DBioSiO_2_.

Figure 9 presents UV-Vis absorption spectra for the undoped diatom biosilica sample (dark blue line) and diatom biosilica doped with cerium (red line). Using UV-Vis spectrophotometric analysis in the range from 250 nm to 900 nm, a flat absorption profile was observed for the DBioSiO_2_ sample. Such a phenomenon indicates the limited electron activity of diatom biosilica in the studied range.

In contrast to the DBioSiO_2_ sample, the 10%Ce-DBioSiO_2_ sample shows a distinct absorption peak at lower wavelengths. This indicates the presence of characteristic electron transitions associated with CeO_2_ nanoparticles, which introduce new energy levels in the excited energy gap. Light absorption by cerium-doped biosilica begins below 400 nm, characteristic of Ce electron transitions in these areas. The broadband occurring from ~260 nm to ~375 nm (with a peak maximum of 315 nm) may correspond to charge transfer (CT) from the 2*p* oxygen (O^2−^) orbitals to the 4*f* Ce^4+^ orbitals [55,56,57,58]. The Ce 4*f* orbital is located between the valence band of O 2*p* and the conduction band of Ce 5*d*.

**Figure 10:**
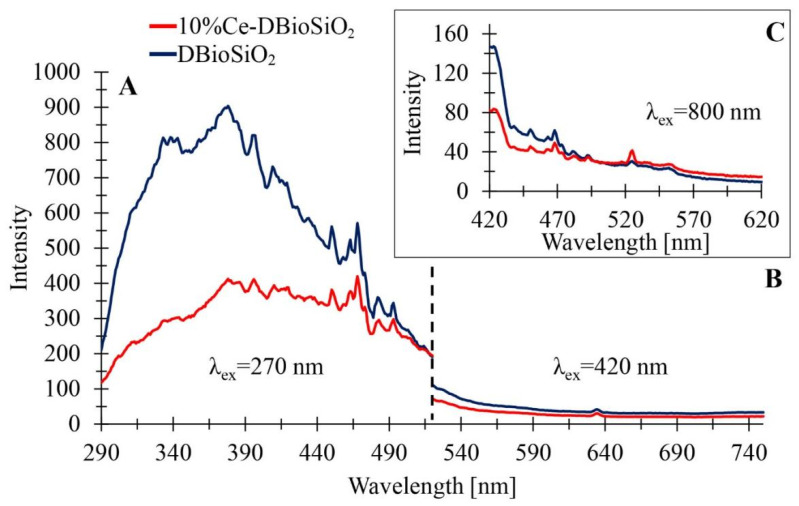
Photoluminescence spectra of samples DBioSiO_2_ and 10%Ce-DBioSiO_2_ presented in the three analysed ranges at excitation wavelengths of (**A**) 270 nm, (**B**) 420 nm, and (**C**) 800 nm.

Furthermore, a band with a maximum of 315 nm provides strong evidence that the CeO2 NPs-containing material is optically and photocatalytically active [57,59]. It is worth noting that as the wavelength increases, the cerium-doped material absorbance value decreases. This behaviour in the UV-Vis range is a characteristic feature of some rare earth oxides [60]. A similar trend can also be observed when examining the emission spectra, as depicted in Figure 10.

Figure 10 presents the emission spectra of the samples of DBioSiO_2_ (dark blue line) and 10%Ce-DBioSiO_2_ (red line), captured at the following three excitation wavelengths: (A) 270 nm, (B) 420 nm, and (C) 800 nm. Figure 10A displays the photoluminescence (PL) spectrum in the wavelength range from 290 to 520 nm, with an excitation wavelength of 270 nm. In contrast, Figure 10B shows the PL spectra in the wavelength range from about 520 to 750 nm, where the excitation wavelength is 420 nm. The emission of radiation in the violet–blue and green visible light region, evident in these spectra, can be attributed to defects associated with the silica matrix: non-bridging oxygen hole centres or oxygen vacancies present [61,62,63]. Additionally, this emission could result from the presence of oxygen vacancies in the structure of CeO_2_. In the cubic structure of ceria, the oxygen ions are not densely packed, forming numerous oxygen vacancies. This is in line with the Kröger–Vink notation presented in Equation (2) [59], as follows:

(2) $${4Ce}_{Ce}+O_{O}\to {2Ce`}_{Ce}+V_{O**}+\left(\frac{1}{2}\right)O_{2}\left(g\right)$$

where Ce_Ce_ denotes the Ce^4+^ crystal lattice dedicated to Ce, Ce`_Ce_ represents the Ce^3+^ ion on the Ce lattice site, O_O_ corresponds to O^2−^ on the crystal lattice dedicated to O, and V_O**_ denotes the neutral oxygen vacancy site. Removal of an oxygen atom from cerium dioxide (CeO_2_) leads to the appearance of electron pairs trapped in the oxygen vacancy, forming so-called F centres. The oxygen defect states in the CeO_2_ structure are located just below the Ce 4*f* energy level. Kröger–Vink notation indicates the presence of Ce^3+^ ions at grain boundaries, acting as hole traps, while oxygen vacancies are electron traps, generating emission peaks in the blue and green regions [59]. Figure 10C shows a spectrum ranging from 420 to 620 nm, with an excitation wavelength of 800 nm. It indicates an up-conversion process in which the absorption of two or more photons of lower energy results in the emission of a single photon of higher energy. A noticeable decrease in emission intensity at longer wavelengths is characteristic of this phenomenon [60]. The green range’s most intense emission peak implies converting a longer-wavelength (red) photon to a shorter-wavelength, higher-energy photon.

In the relative method, the fluorescence quantum yield of a sample is usually determined by comparing its fluorescence intensity with that of a standard of known quantum yield. This procedure requires appropriate matching of the absorption spectra of the sample and the standard, as well as similar measurement conditions (e.g., type of solvent, pH value, or temperature). Substances with stable and well-characterized fluorescence are used as standards to obtain the most accurate values of the samples’ quantum yield. Table 1 summarises the data needed to calculate the fluorescence quantum yield using the relative method for 10%Ce-DBioSiO_2_. The computed value of the fluorescence quantum yield was 1.15%. The luminescence efficiency of rare earth ions is significantly reduced when defects are present in the crystal lattice. The phenomenon of permanent luminescence is a recent application of rare earths based on lattice defects [61].

## 4. Conclusions

For the first time, three-dimensional micro-nanostructured amorphous biosilica functionalised with cerium oxide nanoparticles has been prepared using a biosynthesis method involving unicellular microalgae (diatoms) capable of metabolically absorbing and depositing cerium from grow medium on their silica exoskeleton (diatom biosilica).

They obtained a composite (Ce-DBioSiO_2_) containing cerium 10 wt.%, which combines the unique structural and photonic properties of diatom biosilica and the functionality of immobilised CeO_2_ nanoparticles. Diatom biosilica doped with cerium depicts the uniform distribution of the created nanoclusters (around 100 nm) of cerium nanoparticles (10–25 nm) exhibiting an irregular, quasi-cubic shape. The XRD analysis results confirmed the presence of crystalline forms of CeO_2_NPs with a slight crystal lattice deformation, which probably could have been caused by the purification of biosilica from biomass using hydrogen peroxide. The hydrogen peroxide treatment of the diatom biomass may also have resulted in a significant (twofold) increase in the hydration of the cerium-doped biosilica compared to pure biosilica, detected by thermogravimetric analysis.

The Ce-DBioSiO_2_ composite exhibits an affinity for intense absorption in the wide wavelength range of 250–500 nm, intense fluorescence in the violet–blue region under UV irradiation, and intense up-conversion fluorescence in the violet region under 800 nm near-infrared excitation with a xenon lamp at room temperature in an ambient atmosphere.

## Figures and Tables

**Table 1:** Summary of data necessary to calculate the fluorescence quantum yield using the relative method for the 10%Ce-DBioSiO_2_ sample.

Name	$n_{{fl}_{sample}}$	$n_{{fl}_{reference}}$	${IF}_{sample}$	${IF}_{reference}$	$n_{sample}^{2}$	$n_{reference}^{2}$
**10%Ce-DBioSiO_2_**	0.01154	0.59	5266.824	276,481.801	1.859	1.812

## Data Availability

Data will be made available on request.
